# Trends in US home food preparation and consumption: analysis of national nutrition surveys and time use studies from 1965–1966 to 2007–2008

**DOI:** 10.1186/1475-2891-12-45

**Published:** 2013-04-11

**Authors:** Lindsey P Smith, Shu Wen Ng, Barry M Popkin

**Affiliations:** 1 Department of Nutrition, University of North Carolina, Chapel Hill, CB # 8120 University Square, 123 W. Franklin Street, Chapel Hill, North Carolina, NC 27516-3997, USA; 2 The Carla Chamblee Smith Distinguished Professor of Global Nutrition, Department of Nutrition, Gillings School of Global Public Health and School of Medicine, CB # 8120 University Square, Chapel Hill, NC 27516-3997, USA

**Keywords:** Food preparation, Cooking, Diet, Obesity, Low income

## Abstract

**Background:**

It has been well-documented that Americans have shifted towards eating out more and cooking at home less. However, little is known about whether these trends have continued into the 21^st^ century, and whether these trends are consistent amongst low-income individuals, who are increasingly the target of public health programs that promote home cooking. The objective of this study is to examine how patterns of home cooking and home food consumption have changed from 1965 to 2008 by socio-demographic groups.

**Methods:**

This is a cross-sectional analysis of data from 6 nationally representative US dietary surveys and 6 US time-use studies conducted between 1965 and 2008. Subjects are adults aged 19 to 60 years (n= 38,565 for dietary surveys and n=55,424 for time-use surveys). Weighted means of daily energy intake by food source, proportion who cooked, and time spent cooking were analyzed for trends from 1965–1966 to 2007–2008 by gender and income. *T*-tests were conducted to determine statistical differences over time.

**Results:**

The percentage of daily energy consumed from home food sources and time spent in food preparation decreased significantly for all socioeconomic groups between 1965–1966 and 2007–2008 (*p* ≤ 0.001), with the largest declines occurring between 1965 and 1992. In 2007–2008, foods from the home supply accounted for 65 to 72% of total daily energy, with 54 to 57% reporting cooking activities. The low income group showed the greatest decline in the proportion cooking, but consumed more daily energy from home sources and spent more time cooking than high income individuals in 2007–2008 (*p* ≤ 0.001).

**Conclusions:**

US adults have decreased consumption of foods from the home supply and reduced time spent cooking since 1965, but this trend appears to have leveled off, with no substantial decrease occurring after the mid-1990’s. Across socioeconomic groups, people consume the majority of daily energy from the home food supply, yet only slightly more than half spend any time cooking on a given day. Efforts to boost the healthfulness of the US diet should focus on promoting the preparation of healthy foods at home while incorporating limits on time available for cooking.

## Introduction

American diets have shifted towards decreased nutrient density [1] with less than 20 percent meeting USDA guidelines for a healthy diet, including fruits, vegetables, whole grains and low-fat dairy [2]. US consumers increasingly consume foods from away-from-home sources including fast food, cafeterias, and restaurants [3-7]. In fact, one recent paper showed that for children, half of all energy from fast food is consumed at home [4], demonstrating that even foods consumed within the home are not necessarily home-cooked. Alongside an increase in eating out, people spend less time in food preparation, with an approximate halving of time for women and a small increase for men [8-12], a trend which continued into the 21st century [12,13]. Unsurprisingly, lack of time is reported as a major barrier to preparing nutritious meals [14-16], prompting people to “buy” time through the purchase of convenience foods [17] which are often sold ready-to-eat or requiring minimal preparation. The shift towards increased grazing [18,19] and snacking [20] also decreases time spent cooking, as people reach for portable pre-packaged snacks instead of eating meals.

This well-documented shift towards eating away from home has created a movement to focus on fast food and other chain restaurants as the target of public health initiatives combating obesity. However, few studies have examined whether these trends towards eating out more and cooking less have continued in recent years. There is no evidence to suggest that the trend towards increased away-from-home food consumption should continue indefinitely, and it is possible that US consumers have already reached a peak in terms of how much eat out. A leveling off of this trend could have implications for where efforts for improving the US diet and reducing excess energy intake are directed.

Although concern amongst public health scholars and advocates has often centered on fast food and other away-from-home foods, efforts to boost consumption of healthy home-cooked foods have become increasingly common across the US Programs include the Supplemental Nutrition Assistance Program (SNAP) Healthy Incentives Pilot aimed at increasing purchase of fruits and vegetables and the Women, Infants, and Children (WIC) Farmer’s Market Nutrition program, which provides coupons for the purchase of locally grown produce [21-23]. In both the UK and the US, promotion of home cooking has been viewed as a major strategy to reduce obesity [24-28]. However, these initiatives assume that if consumers are able to purchase healthy foods, they can and will prepare them at home. Little is known about how frequently or how much time Americans spend cooking food in the home, especially among demographic groups targeted by these food assistance programs [29-31].

This study aims to evaluate 1) whether US adults continue to increase away-from-home food consumption or if this trend has leveled off and 2) when people do eat at home, how likely they are to cook and how much time they spend cooking, especially amongst low-income consumers. We examine trends in home food consumption and home food preparation by linking two sets of nationally representative cross-sectional surveys from 1965–1966 to 2007–2008. Using dietary data, trends in the source of people’s food were examined in relationship to mean total daily energy intake. Time-use surveys were utilized to evaluate changes in time allocation for cooking, as well as to examine the role of gender and income.

## Data and methods

### Dietary data

This study used data on 38,565 individuals aged 19 to 60 years from 6 nationally representative cross-sectional nutrition surveys: 4,114 participants from the 1965–1966 Household Food Consumption Survey (HFCS); 12,935 participants from the 1977–1978 Nationwide Food Consumption Survey (NFCS); 7,750 participants from the 1989–1991 Continuing Survey of Food Intakes by Individuals (CSFII); 6,894 participants from the 1994–1996 CSFII; 3,138 participants from the 2003–2004 National Health and Nutrition Examination Survey (NHANES); and 3,734 participants from the 2007–2008 NHANES (Table 1). Detailed methodology pertaining to each survey has been published previously [32-37]. We utilize the first day of dietary recall to provide comparable measures over the full time period studied.

**Table 1:** Characteristics of US adults from national nutrition and time use surveys, 1965-1966 to 2007-2008^*^

**National nutrition surveys**											
	**HFCS**	**NFCS**		**CSFII**		**CSFII**		**NHANES**		**NHANES**	
	**1965- 1966**	**1977-1978**		**1989-1991**		**1994-1996**		**2003-2004**		**2007-2008**	
	**(n=4,114)**	**(n=12,935)**	***p***^***a***^	**(n=7,750)**	***p***	**(n=6,894)**	***p***	**(n=3,138)**	***p***	**(n=3,734)**	***p***
Gender											
Male	43.3%	41.1%	0.104	48.1%	<0.001	49.3%	<0.001	49.1%	<0.001	48.3%	<0.001
Age group											
19 - 30	28.0%	36.3%	<0.001	32.4%	0.167	30.1%	0.640	29.5%	1.000	28.6%	1.000
31 - 40	26.3%	23.6%	0.0243	30.2%	0.007	30.1%	0.002	24.1%	0.804	22.7%	0.048
41 - 50	25.6%	19.8%	<0.001	21.9%	0.006	23.7%	0.249	24.5%	1.000	26.2%	1.000
51 - 60	20.1%	20.3%	1.000	15.5%	<0.001	16.1%	0.001	21.9%	1.000	22.5%	0.437
Income^†^											
Low	21.0%	13.7%	<0.001	12.8%	<0.001	15.3%	0.005	22.9%	1.000	22.7%	1.000
Middle	52.7%	26.7%	<0.001	28.5%	<0.001	28.9%	<0.001	25.9%	<0.001	26.0%	<0.001
High	26.3%	49.6%	<0.001	58.7%	<0.001	55.8%	<0.001	51.2%	<0.001	51.3%	<0.001
**National time use surveys**											
	**MCTRP**	**AUTP**		**AUTP**		**NHAPS 1992–1994 and NTDS**		**ATUS**		**ATUS**	
	**1965- 1966**	**1975-1976**		**1985-1986**		**1994-1995**		**2003-2004**		**2007-2008**	
	**(n=1,888)**	**(n=3,190)**	***p***^***b***^	**(n=2,391)**	***p***	**(n=6,291)**	***p***	**(n=24,382)**	***p***	**(n=17,282)**	***p***
Gender											
Male	47.5%	46.7%	1.000	46.7%	1.000	45.8%	1.000	48.9%	1.000	49.5%	0.556
Age group											
19 - 30	32.3%	35.1%	0.305	34.3%	0.942	31.1%	1.000	25.8%	<0.001	26.2%	<0.001
31 - 40	22.4%	25.5%	0.062	29.7%	<0.001	29.7%	<0.001	27.0%	<0.001	25.6%	0.009
41 - 50	26.3%	18.8%	<0.001	19.1%	<0.001	24.3%	0.428	26.5%	1.000	26.5%	1.000
51 - 60	19.0%	20.6%	1.000	16.9%	0.442	14.9%	<0.001	20.6%	0.549	21.7%	0.046
Income status											
Low	15.9%	9.9%	<0.001	14.5%	1.000	30.5%	<0.001	21.5%	<0.001	19.9%	<0.001
Middle	52.4%	54.2%	1.000	51.1%	1.000	34.4%	<0.001	49.9%	0.208	46.8%	<0.001
High	31.6%	35.9%	0.019	34.4%	0.374	35.1%	0.427	28.6%	0.049	33.3%	0.867

^*^ Abbreviations: HFCS is the Household Food Consumption Survey, NFCS is the Nationwide Food Consumption Survey, CSFII is Continuing Food Intakes by Individuals, and NHANES is National Health and Nutrition Survey. MCTBRP is the Multinational Comparative Time-Budget Research Project, AUTP is American’s Use of Time Project, NHAPS and NTDS are the National Human Activity Pattern Survey and National Time Diary Study, respectively, and ATUS is the American Time Use Study. Percentages have been adjusted to be nationally representative.

^†^Income distributions for the dietary and time use surveys cannot be directly compared as income categories in the diet surveys reflect the ratio of family income to poverty (≤130%, 130% to 300%, and ≥ 300%) while income categories in the time use surveys reflect the quantile of respondent income (lowest 25%, middle 50%, and highest 25%).

^a^ Different from the HFCS 1965–1966 (proportions testing).

^b^ Different from the MCTRP 1965–1966, (proportions testing).

### Categorization of food intake

For all diet surveys, the proportion of energy consumed from home and away food sources for adults was determined using food source and eating location variables. Because this study is concerned primarily with the source of food rather than where the food was eaten, location of consumption is considered only when it is difficult to ascertain the source of food without additional information. To allow comparability across surveys, “home” food sources include any food that is designated as purchased from a store, convenience store, or grocery/deli [4]. Lack of information, including whether food from the store was prepared at home or fully prepared at the store, limited analysis of this component. Foods that were not from the home or the store were considered as away-from-home food sources, including schools, cafeterias, restaurants, and fast food. The proportion of daily energy consumed from home sources was calculated for each participant.

Participants were excluded if the energy content of a food or the source of an energy-containing food was missing. Of participants with dietary and demographic data, the percentage of participants excluded are 10% in 1965–1966 HFCS, 3.2% in 1977–1978 NCFS, 0.6% in the 1989–1991 CSFII, 1.8% in the 1994–1996 CSFII, 0.3% in 2003–2004 NHANES, and 0.2% in the 2007–2008 NHANES.

### Time use data

The American Heritage Time Use Study is a cross-national harmonized dataset of time-use surveys with identically recoded variables [38]. The present study utilizes data on 55,424 adults aged 19 to 60 years from 6 time use studies of US adults: 1,888 participants from the 1965–1966 Multinational Comparative Time-Budget Research Project (MCTRP); 3,190 participants from the 1975–1976 American’s Use of Time Project (AUTP); 2,391 participants from the 1985 AUTP; 5,395 from the 1992–1994 National Human Activity Pattern Survey (NHAPS) and 896 from the 1994–1995 National Time-Diary Study (NTDS); 24,382 from the 2003–2004 American Time Use Study (ATUS) and 17,282 from the 2007–2008 ATUS (Table 1). Participants from the 1992–1994 NHAPS and the 1995–1995 NTDS were pooled for analysis due to overlapping time periods (n=6,291). The analysis sample was restricted to adults aged 19 to 60 to capture adults during prime working ages and to exclude retirees.

The methodological details of these studies have been published previously [39]. All surveys were weighted to balance the distribution of age and sex groups in relation to the Census distribution at the relevant time and to provide an even distribution of days of the week [40].

### Coding and categorization of time use diaries

Diaries were excluded if they did not meet any of the following criteria: 1) had ≥91 min of main activity time after imputation, 2) had ≥ seven time episodes, 3) included ≥ two of four basic activities (sleep/rest, eating/drinking, personal care, and travel/exercise), or 4) included information on age and sex. Of respondents age 19 to 60 years, 0.1% were excluded in the 1965–1966 MCTRP, 2.2% in the 1975–1976 AUT, 1.6% in the 1985–1986 AUT, 3.1% in the 1992–1995 NHAPS/NTDS, 2.8% in the 2003–2004 ATUS, and 2.6% in the 2007–2008 ATUS.

Time spent in food preparation was the sum of time spent preparing food and cleaning up after food preparation. If a participant reported >0 min of time spent in food preparation or food-related cleaning activity, they were considered to have prepared food.

This study analyzed public use data with no personally identifiable information. No institutional review board review was required. Consent was obtained for all participants by the institutions initially responsible for collecting the data.

### Statistical analysis

Demographic variables considered in the analysis included gender, age group, and income level. In the food intake analysis, income was categorized into low, middle, and high income groups if the ratio of family income to poverty was ≤130%, 130% to 300%, and ≥ 300%, respectively. In the time use analysis, income reflects the annual household income recorded in quantiles: lowest 25%, middle 50%, and highest 25%. No continuous income data was available in the time use data set, precluding categorization of income into groups based on family income to poverty for this part of the analysis. Statistical analysis was conducted in 2011 and performed using Stata (Version 12, 2012, StataCorp, College Station, TX). Using survey commands to account for survey design and adjust results to be nationally representative, *t*-tests were conducted to test differences between ratios of the means for each survey year with a two-sided significance level of ≤0.01.

## Results

Demographic characteristics of participants in the nutrition and time use surveys are presented in Table 1.

### Energy intake by food source

Total energy intake and energy consumed from home-source food by gender from HFCS65 to NHANES07 are shown in Additional file 1: Table S1. From 1965–1966 to 2007–2008, total mean daily energy intake increased by 738 kJ/day amongst females (*p* ≤ 0.001), and but did not change significantly for men. Amongst males and females, the percentage of energy consumed from home-source food decreased 24.5% and 23.9% respectively between 1965–1966 and 2007–2008 (*p* ≤ 0.001). In absolute terms, these surveys show a fairly consistent decrease in energy consumed away-from-home until 1994–1996, with virtually no subsequent change in energy consumed away-from home for men or women from 1994–1996 to 2007–2008 .

**Figure 1:**
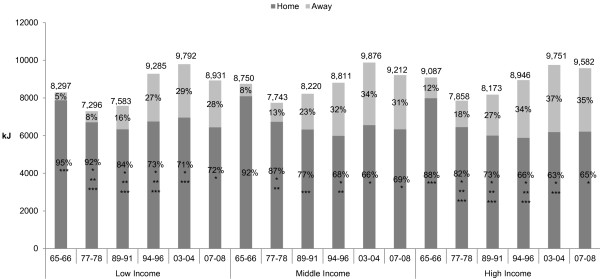
**Daily energy intake of US adults by food source, 1965-1966 to 2007-2008.** Data sources: Household Food Consumption Survey (HFCS) of 1965–1966 (n=4,114), Nationwide Food Consumption Survey (NFCS) of 1977–1978 (n=12,935), Continuing Survey of Food Intakes by Individuals (CSFII) of 1989–1991 (n=7,750), CSFII of 1994–1996 (n=6,894), National Health and Nutrition Examination Survey (NHANES) of 2003–2004 (n=3,138), and NHANES of 2007–2008 (n=3,734). ^*^ Percentage of calories eaten from home sources differed significantly from HFCS 1965–1966, *p* ≤ 0.01 (t-test). ^**^ Percentage of calories eaten from home sources differed significantly from previous survey, *p* ≤ 0.01 (t-test). ^***^ Percentage of calories eaten from home sources differed significantly between low and high income groups for relevant survey year, *p* ≤ 0.01 (t-test).

Trends in total energy intake and energy consumed from home food sources by income group are shown in Figure 1. Overall daily energy increased for all income groups, with total energy increasing by 634 kJ/day in the low income group (*p*= 0.019), 462 kJ/day in the middle income group (*p* = 0.066), and 495 kJ/day in the high income groups (*p* = 0.035). Across all three income groups, consumption from home food sources decreased by approximately 23% from 1965–1966 to 2007–2008 (*p* ≤ 0.001), with the majority of decline occurring prior to 1994–1996 and no significant change occurring during the 2000’s. The lowest income group consumed the highest proportion of home-source food across all years, while the highest income group consumed the least amount of food from home sources across all years.

### Food preparation

Prior to 2003, the sample of adults had a slightly higher proportion of men and lower proportion of low income Americans than the US population. The proportion of men who cooked increased from 29% in 1965–1966 to 42% in 2007-2008(*p* ≤ 0.001), and of those who cooked, time spent cooking increased from 37.4 min/day to 45.0 min/day (*p* ≤ 0.001) (Table 2). For women, the proportion of women cooking declined from 92% in 1965–1966 to 68% in 2007–2008 (*p* ≤ 0.001), and those who did cook showed a decrease in time spent from 112.8 min/day in 1965–1966 to 65.6 min/day in 2007–2008 (*p* ≤ 0.001) (Table 2).

**Table 2:** Trends in Time Spent Cooking for US adults from 1965–1966 to 2007-2008^*†^

**Proportion cooking (%)**													
	**MCTRP**		**AUTP**		**AUTP**		**NHAPS/NTDS**		**ATUS**		**ATUS**		**Change**
	**1965-1966**		**1975-1976**		**1985-1986**		**1992-1995**		**2003-2004**		**2007-2008**		**1965-2007**
Gender	%	SE^‡^	%	SE	%	SE	%	SE	%	SE	%	SE	%
Male	28.6	1.6	29.1	1.3	46.8^ab^	1.6	38.3^a^	0.9	37.9 ^a^	0.5	41.7^ab^	0.6	+13.7
Female	92.3	0.8	88.4^ab^	0.8	84.7^ab^	1.1	67.3^ab^	0.8	69.0^a^	0.4	67.7^a^	0.6	−24.6
Income													
Low	67.6	3.2	69.8^c^	3.2	65.5	3.0	58.1	3.2	55.6^a^	0.8	55.6^a^	1.1	−12.0
Middle	62.7	1.6	61.7	1.4	67.1^b^	1.5	53.1^ab^	3.0	53.1^a^	0.5	53.6^a^	0.7	−9.1
High	59.3	2.1	58.2^c^	1.7	68.4^ab^	1.8	49.6^ab^	3.0	53.9	0.7	56.4^b^	0.8	−2.9
**Mean time spent cooking, of those cooking (min/day)**													
Gender	min/day	SE	min/day	SE	min/day	SE	min/day	SE	min/day	SE	min/day	SE	min/day
Male	36.7	2.1	37.8	1.8	36.1	1.6	39.8	1.2	43.0^ab^	0.7	45.0^a^	0.9	+8.3
Female	112.8	2.2	100.6^ab^	2.0	82.8^ab^	2.1	64.7^ab^	1.3	67.1^ab^	0.6	65.6^ab^	0.8	−47.2
Income													
Low	98.7	5.1	85.8	5.2	73.4^a^	4.5	57.6^ab^	3.9	63.5^ac^	1.2	64.0^ac^	1.7	−34.7
Middle	98.0	2.8	83.6^ab^	2.4	68.5^ab^	2.3	58.8^a^	4.0	57.0^a^	0.8	55.5^a^	0.9	−34.5
High	92.6	3.8	91.9	3.3	65.9^ab^	3.1	63.4^a^	5.4	55.8^ac^	0.9	56.5^ac^	1.0	−36.1

^*^ Data sources include Multinational Comparative Time-Budget Research Project (MCTRP) of 1965–1966 (n=1,888), American’s Use of Time Project (AUTP) of 1975–1976 (n=3,190), AUTP of 1985–1986 (n=2,391), National Human Activity Pattern Survey (NHAPS) of 1992–1994 and National Time Diary Study (NTDS) of 1994–1995 (n=6,291), American Time Use Study (ATUS) of 2003–2004 (n=24,382), and ATUS of 2007–2008 (n=17,282). Percentages and mean time spent cooking are adjusted to be nationally representative.

^†^ Food preparation includes cooking and food preparation-related cleaning activities.

^‡^ SE= standard error.

^a^ Mean differed significantly from MCTRP 1965–1996, *p* ≤ 0.01 (*t* -test).

^b^ Mean differed significantly from previous survey, *p* ≤ 0.01 (*t* -test).

^c^ Mean differed significantly between high and low income groups for relevant year, *p* ≤ 0.01 (*t* -test).

Fewer people cooked in 2007–2008 compared to 1965–1966 for all income groups, although the low income groups showed the largest decline in the proportion cooking, from 67% in 1965–1966 to 56% in 2007–2008 (*p* ≤ 0.001) (Table 2). All income groups showed similar declines in the amount of time spent cooking by those who cooked, with low, middle and high income groups showing a decline of 35–36 min/day from 1965-1966 to 2007–2008 (*p* ≤ 0.001). For all groups, the largest declines in time spent cooking occurred between 1965 to 1992, with declines of 29.2, 39.2 and 41.1 min/day for high, middle, and low income groups respectively. After 1992, decline in time spent cooking leveled off, with the low income group actually increasing cooking time from 57.6 min/day in 1992 to 64.0 min/day in 2007–2008, and the middle and high income groups showing declines of 3.3 min and 6.9 min/day respectively. There were no significant differences in the amount of time spent cooking between low and high income groups except in 2003–2004 and 2007–2008, when those who cooked in low income groups reported 63.5 min/day and 64.0 min/day respectively, compared to 55.8 min/day and 56.5 min/day respectively in the high income group (*p* ≤ 0.001). Time reported in food preparation tended to be clustered around 15 minute intervals at 0, 15, 30, 45, and 60 min as expected due to clumped reporting.

## Discussion

This study showed important shifts in home food consumption and food preparation time patterns. The frequency of eating away-from-home foods increased for all groups from 1965–1966 until 2007–2008, consistent with previous reports [4,6], yet home food sources remain the top source of daily energy across all socio-demographic groups. The overall amount of time spent in food preparation has decreased, as fewer people cook per day and those who cook spend less time on cooking. However, the key finding of this study is that the rate of relative decline in both home food consumption and time spent in food preparation appears to have plateaued in the mid-1990s, with little additional decrease occurring in later years.

When considered together, the dietary and time use data suggest several distinct trends. First, the lack of change in eating out and in cooking during the late 1990’s and early 2000’s suggests that US adults have achieved a stable pattern where roughly a two-thirds of daily energy are consumed from home sources, with the remaining third coming from away-from-home foods, including fast food and restaurants. Given the stability of this trend over the past 20 years, it seems unlikely that US adults will show further increases in eating out.

Second, although the home food supply has remained the top source of daily energy, only slightly more than half of US adults cook foods on a given day. This pattern could emerge as the result of several possibilities. First, because the time use data does not account for activities of other household members, it is possible that those who do not cook are being cooked for by other members of the household. However, given that the proportion of people who cook and the time they spend cooking has declined across all groups, cooking by other household members cannot entirely account for the difference between home food consumption and home cooking. More likely, more people are relying upon ready-to-eat foods that require no preparation. The category of foods requiring no preparation ranges from raw produce (apples, carrots) to snack foods (chips, cookies) to pre-prepared meals from the grocery store. Similarly, the decrease in time spent in food preparation suggests that when people do cook, they are relying more heavily on packaged and convenience foods (e.g., boxed flavored rice, pasta sauce jars, frozen pizzas), which are faster to prepare [14-17]. Future research is needed to better understand how much people cook from scratch, or if “home cooking” consists mostly of heating up pre-processed foods.

This shift from food prepared at-home to increased consumption of convenience/easy-to-prepare and away-from-home foods may have important nutritional implications. Cross-sectional and longitudinal studies have shown that AFH foods have been associated with increased energy intake and decreased nutritional quality [5,7,41-44], as well as increased weight gain [45,46]. In contrast, eating foods prepared from scratch is associated with increased intakes of fruits, vegetables and whole grains [14,47,48]. Increased cooking has also been linked to improved overall health [49], a decrease in BMI [13,50] and improved survival [51].

### Gender differences

While both men and women increased consumption of away-from-home foods from 1965–1966 to 2007–2008, men nearly doubled their overall cooking time, while women more than halved the amount of time spent in food preparation activity. This finding is supported by studies documenting shifts in gender roles, changes in household demographic composition, and the increased education, labor force experience, and occupational attainment of women [10,11,18] resulting in less time spent in food preparation [10-12]. This trend has occurred simultaneously with increases in processed prepared meals, convenience foods, and away-from-home-eating [52-55]. However, women continue to spend more than twice the amount of time cooking than men, suggesting that traditional attitudes towards responsibility for household food preparation persist.

### Income differentials

The percent of daily energy from home food sources decreased by similar amounts across all income groups, but low income individuals consumed a higher proportion of food from home sources across all years. Time spent in food preparation decreased across all three income groups, with the distributions of time spent cooking between income groups becoming more similar to each other in 2007–2008 than in 1965–1966 as fewer people cooked and spent less time cooking. Low income participants spent the most time in food preparation in later years, but showed the greatest decline in proportion preparing food.

Despite consuming 72% of daily energy from home food sources, lower income individuals appear to be increasingly less likely to cook, suggesting a greater reliance upon foods that requiring little preparation rather than increased eating out. A shift towards processed, packaged foods eaten at home is consistent with Drewnowski’s work demonstrating that energy-dense diets comprised of refined grains, added sugars, and added fats cost less than fresh fruits and vegetables, meats, and fish [56], and this price disparity between energy-dense foods and more healthful foods has increased over time [57]. {Monsivais, 2010 #163, [58]} These results show that in addition to cost, time may represent a major barrier to the preparation of raw produce, lean proteins, and whole grains for low income groups.

Indeed, previous studies have shown that time is a main barrier for cooking healthy foods amongst low-income individuals [59] and may prohibit beneficiaries of food assistance programs such as SNAP from meeting healthy meal targets [60-62]. Low-income adults may especially feel the burden of time scarcity and reduce food preparation time [63,64]. Constraints of lower status jobs such as working multiple jobs, long hours, shift scheduling, and overtime pose barriers for low-income adults to prepare meals at home [65].

Lack of cooking knowledge, confidence, and skills can also limit at-home preparation of healthy meals [66,67] and may explain some of the trend towards decreased cooking. Historically, education on food and cooking has been an integral part of the US public school curricula. The Smith-Hughes Act of 1917 provided funding for the training of teachers home economics [68], with widespread provision of home economics classes in middle and elementary schools for much of the 20th century. However, participation in home economics classes in most US schools has declined over recent decades [69], and although this decline occurred after the largest decrease in cooking had already taken place, this trend suggests that decreased cooking confidence and skills may have contributed to decreased cooking amongst young adults in the 1990’s and 2000’s. Although this decrease in formal home economics education in the 1990’s occurred primarily after the largest decline in cooking had already happened, this decline likely remains an important contributor to decreased cooking amongst young adults in the 1990’s and 2000’s. Similarly, informal mechanisms of cooking education such as teaching at home by parents or relatives may decline as adults who cook infrequently are unlikely to teach these skills to their offspring. Unfortunately, the current lack of national data on home economics classes and cooking abilities prevents evaluation of cooking skills and knowledge in the US.

### Limitations

While this study uses nationally representative diet and time use surveys, because neither set of surveys accounts for both food intake and time use, the association between food preparation and daily energy cannot be examined. In addition, the diet data do not provide detailed information on whether food from stores is precooked, semi-processed or purchased as raw ingredients. Furthermore, method differences in data collection over time may further hamper trends analysis of at-home nutrient intake [4] and time use. Because the time use surveys did not collect information on secondary activities outside of childcare, it is possible that as people have begun to multi-task more, they are less likely to report cooking as a primary activity. Such changes in reporting could result in an apparent decline in time spent cooking, even if the actual amount of time spent cooking remained the same.

While this analysis did not control for shifts in the population age structure over time, restricting the analysis sample to adults age 19 to 60 helps to reduce the effect of shifts into the elderly population. Moreover, because older adults tend to cook more and eat out less than younger adults, we expect that adjusting for the aging of the population would result in an even larger decline in cooking in more recent years. Similarly, variation in how race/ethnicity was measured across time in the various surveys precluded adjustment for race/ethnicity in this analysis. Certainly, the population distribution of race/ethnicity has certainly shifted from 1965 to 2007, primarily with regards to the rapidly growing Hispanic population. However, because Hispanics tend to cook more often and for longer, we would expect that adjustment for race/ethnicity would further strengthen the observed decline in cooking. Future work should focus on examining how these key sociodemographic factors affect food preparation and consumption patterns.

Although it is clear that packaged goods rather than raw produce and animal-source foods increasingly represent the bulk of store purchases [70-72], possibly the result of increased consumer demand for such products, the exact nature of the cooking process and skills required remains unknown. Further research is required to fully understand how the food preparation process is linked to dietary intake: is it the amount time spent cooking that matters, is it simply the use of healthier ingredients, or is there some quality about the food preparation processes that yields better food choices? Alternately, the link between cooking and health could represent a selectivity issue, with those cooking extensively having better diet quality due to consciousness about nutrition.

The other major limitation is our inability to examine causal effects using this data. As with many other changes in global eating patterns [73], the underlying cause of the vast shift towards decreased cooking and increased consumption of processed foods [74,75], is partially attributable to demographic shifts including increased female labor force participation, delayed marriage, and smaller family size [10] and as well as food-related societal changes, including the diffusion of labor-saving technology [76], the declining occurrence of family meals [77] and increased snacking [20]. However, with existing data, it is not possible to determine how much these dietary changes are also linked with shifts in marketing and promotion of a lifestyle that includes fast food and processed foods as ways to reduce time spent cooking.

## Conclusion

This study demonstrates that people are eating out more and spending less time cooking, and that these changes appear to have stabilized. These results suggest two possible ways for improving diets in the US: 1) improve the healthfulness of away-from-home foods; and 2) boost people’s ability to prepare healthy foods at home within a short amount of time. In recent years, the public health community has focused on the former strategy, with initiatives ranging from menu labeling to limits on portion sizes of sugar-sweetened beverages purchased at fast food restaurants. However, the present results show that the top source of energy remains food consumed from home sources, and perhaps more importantly, that the shift towards eating out has leveled off. Given that at-home food remains the most important source of Americans’ diet, a more effective approach might seek to boost the healthfulness of foods that people make and consume at home.

One feasible strategy for improving the quality of foods cooked and consumed at home is to promote home-cooking. Yet, these results show that Americans across social classes appear willing to spend only about an hour a day on cooking, or roughly 20 minutes per meal. Considering that this figure has remained stable since the mid-1990’s it seems unlikely that time spent cooking will increase in the near future. SNAP and other programmatic efforts promoting the purchase of produce and preparation of healthy meals must consider limits on time as significant barriers to promoting healthy eating and decreasing fast food intake. This strategy is garnering increased attention by the Institute of Medicine, who recently released a report acknowledging that the nutrition allotment programs need to account the cost–time tradeoff in reaching nutrition goals by applying a time adjustment multiplier to the cost of the Thrifty Food Plan or adjusting the earned income deduction to reflect time pressures [78]. How the USDA handles this adjustment could play a key role in diet quality for these participants: if financial benefits are simply increased so that participants can buy more fully prepared or processed objects, time spent cooking could decrease but so could diet quality. An effort to educate and inform recipients that increased funds can be used to purchase convenient yet healthy items is essential to ensure that increased benefits are not allocated towards increased unhealthy foods.

One possible solution is the return of home economics classes as part of the school curriculum, which could teach young people how to combine healthy convenience items such as canned beans or whole-wheat pasta with foods prepared from scratch [79] to minimize both time and cost. This approach, which has already been initiated in the UK [25-27,80] may be a key strategy for helping Americans reduce weight gain and improve dietary intake. In the US, the *Cooking Matters* program has been rolled out across 40 states, employing 6-week courses to teach children, adults and families at risk of hunger how to purchase and prepare healthy food [81]. Further research is required to determine how home cooking is linked to better dietary intake, and what types of home-cooked foods can maximize both time and health.

### Availability of supporting data

The dietary datasets supporting the results of this article are available in the NHANES repository at http://www.cdc.gov/nchs/nhanes/nhanes_questionnaires.htm. The time use datasets are available with permission from the Centre for Time Use Research at http://www.timeuse.org/mtus/download.

## Abbreviations

HFCS: Household Food Consumption Survey; NFCS: Nationwide Food Consumption Survey; CSFII: Continuing Food Intakes by Individuals; NHANES: National Health and Nutrition Survey; MCTBRP: Multinational Comparative Time-Budget Research Project; AUTP: American’s Use of Time Project; NHAPS and NTDS: National Human Activity Pattern Survey and National Time Diary Study, respectively; ATUS: American Time Use Study, Weighted percentages have been adjusted to be nationally representative; SNAP: Supplemental Nutrition Assistance Program; WIC: Women Infants and Children program.

## Competing interests

The authors declare that they have no competing interests.

## Authors’ contributions

LPS was responsible for data analysis, interpretation of results, and writing the manuscript. BMP provided guidance during all phases of the study, including study conception and design, interpretation of results, and manuscript development. SWN provided guidance during various phases of the study, including study design, data analysis, interpretation of results, and manuscript development. All authors critically reviewed and approved the final manuscript.

## Authors’ information

This work was conducted while LPS was a doctoral student in the Department of Nutrition at the University of North Carolina Gillings School of Global Public Health. BMP is the The Carla Chamblee Smith Distinguished Professor of Global Nutrition and SWN is a research assistant professor in the Department of Nutrition at the University of North Carolina Gillings School of Global Public Health.

## Supplementary Material

Additional files 1: Table S1. US Trends in Cooking Supplemental Text and Tables_ NJ. Click here for file
