# Supplementary material for: Trends in US home food preparation and consumption: analysis of national nutrition surveys and time use studies from 1965–1966 to 2007–2008
Source: Nutr J. 2013 Apr 11;12:45. doi: 10.1186/1475-2891-12-45 (PMC3639863; doi:10.1186/1475-2891-12-45)
Supplement: Additional files 1: Table S1. — US Trends in Cooking Supplemental Text and Tables_ NJ. [file 1475-2891-12-45-S1.doc]

Trends in US home food preparation and consumption: analysis of national nutrition surveys and time use studies from 1965-1966 to 2007-2008

Online Supplement

Supplemental Text

**Methods:**

*Dietary Surveys*

The surveys were self-weighting, multistage stratified area probability samples of the United States population taking place over four seasons. The exception to this methodology was the 1965-1966 HFCS, which was conducted in the spring of 1965 amongst only households with at least one member having a minimum of 10 meals from the home food supply during the week surveyed.

In the 1965-1966 HFCS, participants were asked if the food came from the home food supply. The location of consumption was ascertained only for foods that did not come from the home supply. Foods were considered “from the home” if the respondent indicated that the food was from the home supply, or if the food was not from the home supply, if it was eaten at someone else’s home or at a drug store/deli.

The 1977-1978 NCFS asked respondents if the food came from the home supply and was eaten at home, if the food came from the home supply and was eaten away from home, or if the food was obtained and eaten away from home. Foods were considered from the home if the food item was from the home food supply, regardless of where it were eaten, or if the food item was not from the home supply but was obtained from a grocery or other food store, from a convenience store, or from someone else’s home.

Similarly, the 1989-1991 CSFII asked participants if the food item was eaten at home, brought into the home but eaten away, or never brought into the home. For foods that were brought into the home, participants were asked if the food was from a fast-food place, from Meals on Wheels, or from some other place. Foods were considered from the home if they were brought into the home and not purchased from fast-food or Meals on Wheels, or if they were never brought into the home and were from the store, the supermarket/deli, a convenience store, or someone else’s home.

The 1994-1996 CSFII, 2003-2004 NHANES ,and 2007-2008 NHANES ask separate questions regarding the source of food and eating location. Definitions of food source were consistent across all three surveys. For each survey, home food sources was defined as food that came from the store or food that was grown or caught by the respondent or someone known by the respondent, regardless of where the food was consumed.

*Time Use Surveys*

The 1965-1966 MCTRP used a national multi-stage clustered area sampling of households where one person was of working age and employed in an industry other than agriculture using self-completed 24-hr diaries. The 1975-1976 AUT used stratified, clustered and probability selection within strata to collect 24-hr time-diaries from the same people over four waves: the baseline diary was interview-administered and subsequent diaries completed via telephone. Only the first diary for each individual was used in analysis to ensure comparability with the other surveys. The 1985-1986 AUT used stratified and clustered random-digit dialing to collect 24-hour time diaries via mail-back, telephone, and in-person interview. The 1992-1994 NHAPS and 1994-1996 NTDS also used random-digit dialing to collect 24-hr time diaries via telephone. ATUS in 2003-2004 and 2007-2008 selects participants via a stratified three-state random sub-sample of households that have completed the 8^th^ and final wave of the Current Population Survey (CPS). Respondents complete 24-hr time diaries via computer-assisted telephone interviewing (CATI).

| Supplemental Table 1. Percent of Daily Energy Eaten from Home Sources from 1965-1966 to 2007-2008 by Gender**^*^** | | | | | | | | | | | | | | | | | | | |
| --- | --- | --- | --- | --- | --- | --- | --- | --- | --- | --- | --- | --- | --- | --- | --- | --- | --- | --- | --- |
| Eaten at Home (%) | | | | | | | | | | | | | | | | | | | |
|  | HFCS  1965-1966 | |  | NFCS  1977-1978 | |  | CSFII  1989-1991 | |  | CSFII  1994-1996 | |  | NHANES  2003-2004 | |  | NHANES  2007-2008 | |  | % Change  1965 -2007 |
| Gender | % kJ | SE^†^ |  | % kJ | SE |  | % kJ | SE |  | % kJ | SE |  | % kJ | SE |  | % kJ | SE |  | % |
| Female | 92.8 | 0.4 |  | 86.8^ab^ | 0.4 |  | 76.9^ab^ | 0.9 |  | 68.8^ab^ | 0.6 |  | 65.8^ab^ | 1.0 |  | 68.9^a^ | 1.0 |  | -23.9 |
| Male | 90.6 | 0.6 |  | 82.9^ab^ | 0.5 |  | 74.5^ab^ | 0.6 |  | 66.1^ab^ | 0.9 |  | 65.9^ab^ | 1.0 |  | 66.1^a^ | 0.9 |  | -24.5 |
| Total Daily Energy (kJ) | | | | | | | | | | | | | | | | | | | |
|  | HFCS  1965-1966 | |  | NFCS  1977-1978 | |  | CSFII  1989-1991 | |  | CSFII  1994-1996 | |  | NHANES  2003-2004 | |  | NHANES  2007-2008 | |  | kJ Change  1965 -2007 |
| Gender | kJ | SE |  | kJ | SE |  | kJ | SE |  | kJ | SE |  | kJ | SE |  | kJ | SE |  | kJ |
| Female | 6952 | 92 |  | 6414^ab^ | 50 |  | 6678 | 91 |  | 7186^b^ | 69 |  | 8093^ab^ | 101 |  | 7689^ab^ | 109 |  | +738 |
| Male | 11012 | 174 |  | 9627^ab^ | 83 |  | 9658^a^ | 109 |  | 10786^b^ | 147 |  | 11613^b^ | 163 |  | 11078 | 166 |  | -67 |
| ^*^ Data sources: Household Food Consumption Survey (HFCS) of 1965- 1966 (n=4,114), Nationwide Food Consumption Survey (NFCS) of 1977-1978 (n=12,935), Continuing Survey of Food Intakes by Individuals (CSFII) of 1989-1991 (n=7,750), CSFII of 1994-1996 (n=6,894), National Health and Nutrition Examination Survey (NHANES) of 2003-2004 (n=3,138), and NHANES of 2007-2008 (n=3,734). Proportion of daily energy eaten at home and mean total daily energy are adjusted to be nationally representative.  ^†^ SE= standard error  ^a^ Energy differed significantly from HFCS 1965-1966, *p* ≤ 0.01 (*t*-test) ^b^ Energy differed significantly from previous survey, *p* ≤ 0.01 (*t*-test) | | | | | | | | | | | | | | | | | | | |
